# Determination of the Level of Selected Elements in Canned Meat and Fish and Risk Assessment for Consumer Health

**DOI:** 10.1155/2020/2148794

**Published:** 2020-04-11

**Authors:** Grażyna Kowalska, Urszula Pankiewicz, Radosław Kowalski

**Affiliations:** ^1^Department of Tourism and Recreation, University of Life Sciences in Lublin, Lublin, Poland; ^2^Department of Analysis and Evaluation of Food Quality, University of Life Sciences in Lublin, Lublin, Poland

## Abstract

The objective of the study was to determine the content of cobalt, silver, tin, antimony, lead, mercury, cadmium, arsenic, vanadium, chromium, manganese, nickel, and uranium in canned meat and canned fish by means of ICP-MS apparatus and mercury analyzer. Also, probabilistic risk assessment (non carcinogenic) was estimated by models including target hazard quotient (THQ). It was found that Mn was the element with the highest concentration in the analyzed products, with average concentration of 0.216 mg·kg^−1^ in canned meat and 1.196 mg·kg^−1^ in canned fish. The average contents of other elements were as follows (respectively, for canned meat and fish): Co 0.018 and 0.028 mg·kg^−1^, Ag 0.0386 and 0.0053 mg·kg^−1^, Sn 0.059 and 0.200 mg·kg^−1^, Sb 0.0268 and 0.0377 mg·kg^−1^, Pb 0.202 and 0.068 mg·kg^−1^, Hg 0.00003 and 0.02676 mg·kg^−1^, Cd 0.00496 and 0.0202 mg·kg^−1^, As 0.002 and 0.857 mg·kg^−1^, V 0.0003 and 0.095 mg·kg^−1^, Cr 0.244 and 0.590 mg·kg^−1^, Mn 0.216 and 1.196 mg·kg^−1^, Ni 0.004 and 0.088 mg·kg^−1^, and U < LOQ and 0.047 mg·kg^−1^. The concentration of As was the highest among other toxic elements in canned fish; therefore, the THQ value of this element revealed the highest level amounting up to 0.77576 (THQ_max_).

## 1. Introduction

Food of animal origin is among those products that provide many important nutrients. The food industry employs numerous technologies which allow manufacturing of products with diversified shelf life. Canned products are characterized by a long shelf life, do not need to be kept at low temperature, and do not require special treatment during transport or distribution [1, 2]. The name “canned food” means the food product enclosed in metal cans, glass jars, or plastic containers, the long shelf life of which is ensured through the process of pasteurization and airtightness of the packaging, providing protection against the access of air and contaminants. Some canned foods contain also chemical preserving additives, e.g., sodium nitrate or potassium nitrate [3]. Canned meat and fish represent on average a fairly large share in the food market in Poland. Canned food products, in spite of their taste and nutritive values, can also contain chemical contaminants, the primary source of which is the environment, as well as incorrect technological processing or incorrect packaging [4]. In spite of the implementation of rules of good industrial and agricultural practice in food production, it is not possible to entirely eliminate the presence of chemical contaminants in food due to environmental contamination [5]. Among the numerous contaminants, heavy metals pose a serious threat to human health [6].

Taking into account the above considerations, the objective of this study was to determine the content of selected elements—cobalt, silver, tin, antimony, lead, mercury, cadmium, arsenic, vanadium, chromium, manganese, nickel, and uranium—in canned foods produced from raw materials of animal origin, with particular emphasis on comparing the two most popular groups of canned meats and canned fishes. In addition, the health risk assessment related to the consumption of this product group was determined.

## 2. Materials and Methods

### 2.1. Experimental Material and Sample Preparation

The research material consisted of Polish-made commercial products (Table 1): canned meats (14 assortments from 3 kinds of meat: beef, pork, and chicken) and canned fishes (16 assortments from 6 fish species: tuna fish, sardine, sprat, herring, mackerel, and brown bullhead) purchased from the hypermarket in Lublin (year of production 2017). The content of every can was homogenized. Next, the samples were frozen (−20°C), lyophilized in a Labconco freeze dryer (Model 64132, Kansas City, MO, USA), and stored in a dessicator for further use.

### 2.2. Measurement of Water Content

Water content in fresh and freeze-dried samples was determined with the use of a moisture analyzer (Radwag WPS50SW) after drying samples at 100°C. The result was the average of three measurements.

### 2.3. Determination of Cobalt, Silver, Tin, Antimony, Lead, Cadmium, Arsenic, Vanadium, Chromium, Manganese, Nickel, and Uranium Concentration

Three subsamples derived from the samples taken from every can have been analyzed: about 0.5000 g of sample material was weighed directly into a Teflon vessel, 10 mL of 65% HNO_3_ was added (Suprapur grade, Merck, Germany), and microwave mineralization was performed (Mars 5, CEM Corporation, USA). A blank sample containing only the reagents was attached to every mineralization batch. The microwave mineralization was performed stepwise at 400 W and 363 K (4 min), at 800 W and 393 K (5 min), and at 1600 W and 483 K (6 min). The cooled digested solution was then diluted to 50 mL using high purity deionized water.

### 2.4. ICP-MS Measurements

For determination of Co, Ag, Sn, Sb, Pb, Cd, As, V, Cr, Mn, Ni, and U in the samples, the inductively coupled plasma mass spectrometer ICP-MS 820-MS (Varian, Mulgrave, Australia) with quadrupole mass analyzer has been used. The instrumental conditions for trace elements determination by ICP-MS were as follows: plasma: argon plasma; plasma flow: 18 L min^−1^; auxiliary flow: 1.8 L·min^−1^; stealth gas flow: 0.12 L·min^−1^; nebulizer flow: 0.95 L·min^−1^; sampling depth: 6 mm; RF power: 1.35 kW; pump rate: 0.1 Hz; stabilization delay: 35 s; first extraction lens: 5 V; second extraction lens: 190 V; third extraction lens: 225 V; corner lens: 200 V; left mirror lens: 39 V; right mirror lens: 34 V; bottom mirror lens: 36 V; entrance lens: 1.00 V; fringe bias: −2.90 V; entrance plate: −39 V.

Calibration curve for each element was prepared using the highest purity standard solutions (1000 mg·L^−1^, 99.999%) from Ultra Scientific. The calibration standards for ICP-MS analysis were prepared by diluting the solutions with 1% HNO_3_. The results were expressed in mg·kg^−1^ of fresh matter. The analytical quality was controlled by means of measurement of a blind sample, a double sample, and the certified reference materials: NIST-1577c Bovine Liver, NIST SRM 1548a, and TM 27.3.

Table 2 presented validation parameters obtained during analysis.

### 2.5. Determination of Hg Concentration

Mercury was determined independently using non-flame atomic spectrometry absorption technique (Mercury Analyzer AMA 254, Altec, Czech Republic) according to previously described method [7]. Correct operation of the apparatus was controlled regularly by calibration of standard mercury solutions—NIST-traceable Hg standard solution (Accu Trace Single Element Standard; AccuStandard Inc., New Haven, CT, USA) [7]. The NIST-1577c Bovine Liver was used as the reference material.

### 2.6. Health Risk Assessment

The health risk assessment (noncarcinogenic hazard) related to the presence of heavy metals in analyzed products was performed by using the previously described model [8]. The target hazard quotient (THQ) was used for the calculation of noncarcinogenic hazard of ingestion of heavy metals (1) [8, 9].

(1) $$\begin{array}{c} \text{THQ}=\frac{\left({\text{EF}}_{i}\times {\text{ED}}_{i}\times {\text{MS}}_{i}\times \text{C}\right)}{\left({\text{RfD}\times \text{BW}}_{i}\times \text{AT}\right)}. \end{array}$$

The estimated daily intake EDI (mg analyzed element kg^−1^ body weight day^−1^) was calculated using the following equation [8, 14]:

(2) $$\begin{array}{c} \text{EDI}=\frac{\left({\text{MS}}_{i}\times \text{C}\right)}{{\text{BW}}_{i}}. \end{array}$$

C is the the trace element concentration in canned meat and canned fish (expressed as *μ*g·kg^−1^·w.w. in EDI and as *μ*g kg^−1^ in THQ).

MS_*i*_ is the mass of selected dietary ingested in adults. The average daily consumption of canned products in Poland is as follows: (1) canned meat, 4.5 g·day^−1^ (which corresponds to a portion of about 0.1 canned meat item weighing 300 g week^−1^); canned fish, 11 g·day^−1^ (which corresponds to a portion of about 0.6 canned fish items weighing 120 g week^−1^); (2) canned meat, 43 g·day^−1^ (one canned meat item with a product content of 300 g·week^−1^); canned fish, 17 g·day^−1^ (one canned fish item with a product content of 120 g·week^−1^).

EF_*i*_ is the exposure frequency (365 days·year^−1^ for people who eat canned meat and canned fish seven times a week; 208 days·year^−1^ for people who eat canned meat and canned fish four times a week; 52 days·year^−1^ for people who eat canned meat and canned fish once a week).

ED_*i*_ is the exposure duration: (1) 70 years, equivalent to the average lifetime; (2) 30 years.

BW_*i*_ is the average body weight (70 kg).

AT is the average exposure time for noncarcinogens (365 days·year^−1^ × ED).

When THQ > 1, there is a probability of potentially harmful effects occurring, while at THQ ≤ 1, there is no probability of unfavorable effects [9].

RfD is the heavy metal oral intake reference dose (mg kg^−1^ day^−1^). RfD for cobalt, silver, tin, antimony, lead, mercury, cadmium, and arsenic is 0.0200, 0.005, 63, 0.0004, 0.0036, 0.0003, 0.001, and 0.0003 mg kg^−1^·day^−1^, respectively [10–13].

In order to estimate total target hazard quotient (TTHQ) via multiple heavy metals, the sum of THQ_i_ for individual heavy metal was estimated by (3) [9, 10].

(3) $$\begin{array}{c} \text{TTHQ}=\sum_{i=1}^{n}{\text{THQ}}_{i}. \end{array}$$

### 2.7. Statistical Analysis

Data were analyzed using one-way ANOVA followed by Duncan's test using the SAS statistical system (SAS Version 9.1, SAS Inst., Cary, NC, USA). The significance of all tests was set at *p* ≤ 0.05.

## 3. Results and Discussion

### 3.1. Concentration of Trace Elements in Canned Meats and Fishes

The results of measurements were collected in Tables 3 and 4. Generally, it was demonstrated that concentration of analyzed heavy metals in canned fishes was higher than that in the case of canned meat.

In the canned fishes the highest level of manganese was noted, with a mean value of 1.196 mg·kg^−1^ (from 0.137 mg·kg^−1^ in tuna fish to 2.566 mg·kg^−1^ in sardines), while in the canned meats the level of that element was 0.216 mg·kg^−1^ (from 0.129 mg·kg^−1^ in pork to 0.624 mg·kg^−1^ in chicken). Canned tuna, mackerel, and brown bullhead contained lower levels of Mn compared to sardine, sprat, and herring. The next metal in terms of its content in canned fish is arsenic, with a mean level of 0.867 mg·kg^−1^ (from 0.359 mg·kg^−1^ in brown bullhead to 1.481 mg·kg^−1^ in sardines), while in the canned meats the mean content of As was 0.002 mg·kg^−1^ (from 0.002 mg·kg^−1^ to 0.003 mg·kg^−1^). Chromium was determined in the analyzed canned products at the following levels: 0.590 mg·kg^−1^ in canned fishes, 0.244 mg·kg^−1^ in canned meats. The content of tin in the canned fishes was in the range from 0.018 mg·kg^−1^ in sardines to 1.362 mg·kg^−1^ in brown bullhead, with a mean value of 0.200 mg·kg^−1^, while in the canned meats the mean value was 0.059 mg·kg^−1^ (from 0.005 mg·kg^−1^ in pork luncheon meat to 0.174 mg·kg^−1^ in beef). Among the analyzed assortments, significant differences were noted in the case of mercury–0.02676 mg kg^−1^ in canned fishes (from 0.00610 mg·kg^−1^ in brown bullhead to 0.07840 mg·kg^−1^ in tuna) and 0.00003 mg·kg^−1^ in canned meats (from 0.00001 mg·kg^−1^ to 0.00007 mg·kg^−1^). The content of cadmium in the canned fishes fell in the range from 0.0033 mg·kg^−1^ (sardine, brown bullhead) to 0.0754 (tuna), with a mean value of 0.0202, while in the canned meats the content of Cd, in most of the products, was below LOQ and approached 0.02731 mg·kg^−1^ with mean of 0.00496, and it was lower than that in the canned fishes. On average, silver and lead occurred at higher levels in the canned meats than in the canned fishes, i.e., Ag 0.0386 mg·kg^−1^ (canned meats) and 0.0053 m·kg^−1^ (canned fishes), Pb 0.202 mg·kg^−1^ (canned meats) and 0.068 mg·kg^−1^ (canned fishes). In addition, the canned fishes contained higher levels of nickel (0.088 mg·kg^−1^ in canned fishes, 0.004 mg·kg^−1^ in canned meats) and vanadium (0.095 mg·kg^−1^ in canned fishes, 0.0003 mg kg^−1^ in canned meats) compared to the canned meats. The mean content of cobalt and antimony in the analyzed canned meats and fishes was as follows: Co 0.018 and 0.028 mg·kg^−1^, Sb 0.0268 and 0.0377 mg·kg^−1^, respectively. In the canned meats the content of uranium was below LOQ, while in the canned fishes it was in the range from values below LOQ to 0.226 mg·kg^−1^.

### 3.2. Comparison with Reported Literature Values and with International Dietary Standards and Guidelines for Mercury, Arsenic, Cadmium, Lead, Tin, Chromium, Manganese, Nickel, Cobalt, Silver, Vanadium, Antimony, and Uranium

Table 3 presents data concerning the content of mercury, arsenic, cadmium, lead, and tin in the analyzed canned products in relation to literature data and to the maximum allowable residual levels of each heavy metal in the meat and meat products of pork, beef, chicken, and in fishes and fish products, in accordance with the national and international dietary standards and guidelines.

Fishes and fish products constitute a fairly frequent object of research in this area, but there are a few up-to-date reports comparing meat products with fish products. In the case of fish products, there are several papers on level of Hg, As, Cd, and Pb in canned tuna fish (Table 5) [15–17]. Researches pay special attention to this species, as it is a predatory fish and it can accumulate large amounts of heavy metals. Another reason for the research is the high consumption of this food in various countries [18].

Mercury is a toxic metal commonly occurring in the environment due to its extensive applications. This element accumulates in the brain, kidneys, and hair. A too high level of mercury in the organism results in serious poisoning and chronic pathogenic conditions, frequently leading to death [19]. The provisional tolerable weekly intake (PTWI) for inorganic mercury is 4 *μ*g·kg^−1^ of body mass and for the organic form, methylmercury, 1.6 *μ*g·kg^−1^ of body mass [20]. It is important to monitor the level of this element in various food products, especially in fish products which are among the main sources of mercury introduced into the organism along the alimentary pathway. Other authors addressed the issue of mercury contamination of the human population in respect of nutrition, life style, and mercury level in herbal products, cereal products, and tobacco or the contamination of birds of prey in Poland [19, 21–25]. In accordance with the regulation CE 1881/2006 with later revisions, the established maximum allowable level of mercury content in fish products is 0.5 mg·kg^−1^, and 1.0 mg·kg^−1^ in the case of certain specified fish species, e.g., tuna fish [26]. However, there are no upper limits for mercury content in meat products. The analyses performed within the scope of this study demonstrated that canned fish contained statistically significantly higher levels of mercury than canned meat. Both the earlier studies and the results presented in this paper (Table 5) do not indicate excessive levels of Hg in the Polish canned fish and meat products.

Arsenic is a natural component of most soils, which results in its presence in products of plant origin. Unfortunately, in certain parts of the world, e.g., in the region of Bangladesh, drinking water is a source of As. Arsenic compounds find application as catalysts, bactericides, herbicides, fungicides and admixtures to animal feed, corrosion inhibitors, veterinary medicines, tanning agents, and wood protection agents and were even used as first medicines in the treatment of syphilis [27]. The supply of inorganic arsenic to the human organism leads to disturbance in the functioning of the kidneys and the liver, anaemia, disturbance in the functioning of the alimentary tract, and decrease of body mass may result in neoplastic processes. Due to the lack of legal regulations in the EU on limiting the concentration level of arsenic in food, the discussion of the results is difficult [26]. However, because of the high toxicity of that element, many EU member countries introduced the so-called national maximum allowable levels of arsenic which in Poland are as follows: in meat of mammals and poultry 0.20 mg kg^−1^, in liver and kidneys 0.50 mg·kg^−1^, and in fishes 4.0 mg·kg^−1^ [28]. The analyses performed in this study demonstrated that the canned fish products contained statistically significantly higher levels of As than the canned meats, and in addition those levels did not exceed the established limits values.

Cadmium is a dangerous and toxic metal which may migrate to the organism with food. Cadmium contributes to damage to the functioning of renal tubules, causing increased secretion of low-molecular proteins, disturbs the metabolism of calcium and vitamin D, and has a neurotoxic effect and a destructive effect on the bone system. Cadmium intensifies cardiovascular diseases and hypertension, causes damage to the liver, affects the functioning of the sexual glands, and reduces the body resistance. Cadmium causes also inhibition of the absorption of copper, manganese, zinc, and iron by the organism [29]. According to FAO/WHO recommendations, tolerable weekly intake of cadmium by an adult human is 0.4–0.5 mg, and the maximum allowable dose is 60–70 *μ*g per day. In conformance with regulation EC 1881 [26] with revisions, the established maximum allowable level of cadmium in beef (with the exception of the offal), mutton, and pork is 0.05 mg·kg^−1^, but in the offal 0.5 mg·kg^−1^ and in fish meat 0.05 mg·kg^−1^, except for, e.g., sardine and tuna fish, 0.1 mg·kg^−1^, and swordfish, 0.3 mg·kg^−1^ [26]. Like As and Hg, also Cd was found to be at higher concentrations in the canned fishes than in the canned meats, but its levels did not exceed the established limit values.

Lead damages and destroys erythrocytes; reduces resistance; weakens the bones; blocks the nervous system; inhibits the absorption of iodine, necessary for correct functioning of the thyroid gland; forms toxic deposits in the organism, causing numerous disorders and diseases, enzymes, liver; causes the loss of appetite; causes colics and muscle cramps; causes paralysis; damages the kidneys; raises blood pressure; damages the marrow; and disturbs the metabolism of elements essential for human life, i.e., iron, copper, zinc, and selenium [30]. According to regulation EC 1881 with revisions, the established maximum allowable level of lead content in beef (with the exception of the offal), mutton, and pork is 0.1 mg·kg^−1^, but in the offal 0.5 mg·kg^−1^ and in fish meat 0.3 mg·kg^−1^ [26]. In the present study, the concentration of lead in the canned meats was found to be higher than in the canned fishes, but it did not exceed the established limits.

Tin is relatively less toxic than mercury, cadmium, lead, and arsenic. One of the problems related to the possibility of tin liberation from plating of metal containers for food is faulty cans or the presence of an acidic factor (for example, tomato) in the food product, which facilitates the release of the metal to the food. High levels of tin can cause gastric and intestinal irritation and disorders [31]. According to regulation EC 1881 with revision, the established maximum allowable level of tin in food, with the exception of drinks, is 200 mg·kg^−1^ [26]. Our results have shown that the canned fishes contained higher concentrations of Zn than the canned meats; however, those did not exceed the limit values.

Chromium, with an oxidation state of +3, is an essential trace element that is important for human health. It is included in the so-called Glucose Tolerance Factor (GTF), necessary for correct metabolism of glucose. In addition, it plays an important role in the transformations of proteins (for example, it is a component of trypsin) and lipids (especially of cholesterol) and enhances the effect of insulin [27]. However, chromium at +6 state of oxidation reveals a harmful effect on human health even at small concentrations. Those compounds (chromates) show a strong mutagenic and teratogenic effect [32]. Various methods of food preparation and storage can change the content of chromium in food. If food products are stored in metal containers or cans, the content of chromium increases [33]. Manganese is considered to be an element that is essential for human life. It also participates in transformations of hydrocarbons and lipids and has an activating effect on enzymes, especially those that facilitate the absorption of certain vitamins during metabolism. It is also necessary to maintain correct bone structure and plays an important role in the formation of thyroxine, the main hormone produced by the thyroid gland. However, in certain cases it can pose a threat to human health. Oxygen-containing manganese compounds have a strong toxic effect, depending on oxidation state. Symptoms of manganese poisoning are mainly hallucinations, memory loss, and nerve damage [33]. The recommended daily intake of manganese should not exceed levels from 3.0 to 9.0 mg [34]. According to the literature, Cr and Mn contents in canned fish were found to be as follows: in the muscles of fish from Turkey, 0.19–2.80 and 0.08–3.88 mg·kg^−1^ [35]; in canned tuna and mackerel from the USA, Cr 0.0–0.067 and 0.01–0.30 mg·kg^−1^, Mn 0.0–0.001 and 0.0–0.001 mg·kg^−1^ [18]; in canned tuna from Turkey, Cr 1.08 mg·kg^−1^, Mn 0.90 mg·kg^−1^ [36]; in the canned fish samples from Iran, Cr 0.90–1.87 mg·kg^−1^, Mn 1.20–2.70 mg·kg^−1^ [37]; in canned tuna from the Kingdom of Saudi Arabia, 0.0029 for Mn, 0.0005 for Cr [38]; in the canned tuna from Mexico, for Cr 0.02 to 0.65 mg·kg^−1^, 0.07 to 0.38 mg·kg^−1^ in the fresh fish samples [39]; in canned fish from China, Cr 0.08–1.28 mg·kg^−1^ [40]; in canned tuna from the Kingdom of Saudi Arabia, Cr 0.10–0.57 mg·kg^−1^ [41]; in muscles of fresh fish from Iraq, Mn 0.11–1.86 mg·kg^−1^ dry weight, in the muscles of frozen fish species 0.13–4.50 mg·kg^−1^ dry weight, and in canned fish 0.13–0.81 mg kg^−1^ dry weight [42]; in canned tuna from Ghana, Mn 0.001–0.057 mg·kg^−1^ [43]. Cr content in Bangladesh fish ranged from 2.09 to 7.18 mg·kg^−1^, and Mn from 23.23 to 25.65 mg·kg^−1^ [44]. Other authors report that Mn content in various pork products from European Union was from 0.08 mg·kg^−1^ to 2.62 mg·kg^−1^ [45]. In canned luncheon meat from the Kingdom of Saudi Arabia, Mn concentration was determined at 32.67 mg·kg^−1^ in dry weight mg·kg^−1^ [46]. Cr content in canned corned beef from Iraq was from 0.10 to 0.40 mg kg^−1^ (mean 0.22 mg·kg^−1^) and 0.10 mg·kg^−1^ in the canned chicken luncheon samples [47]. The content of Cr in beef from Nigeria was, on average, 1.24 mg·kg^−1^ [48]. Average Cr concentration in pork luncheon meat from India was 0.598 mg·kg^−1^ [49]. The results obtained in this study, i.e., average Cr of 0.590 mg·kg^−1^ and Mn of 1.196 mg·kg^−1^ in canned fish, were at a level not exceeding the range of the literature data.

Practically Ni only occurs as bivalent ion. It is ingested by humans with food, and its majority is excreted from the organism, but that inhaled with atmospheric dust largely accumulates in the lungs and causes damage to the mucous membranes. Nickel is not an essential element, but its deficit inhibits growth and causes a lowering of the level of haemoglobin in blood as well as changes in the epidermis and disturbance in the pigmentation [50, 51]. Deficit of that element impairs also the function of the liver, whereas excessive levels of nickel reduce the levels of other elements in the organism, such as magnesium, manganese, and zinc. From the alimentary tract, nickel is absorbed in the human organism in 10%. In humans, the levels of nickel absorption are very low, of the order of over ten mg·kg^−1^. According to the literature data, the level of Ni in canned fish was as follows: in canned tuna from the Kingdom of Saudi Arabia, 0.09–0.48 mg·kg^−1^ [41]; in the muscles of fish from Turkey, 0.03–0.63 mg·kg^−1^ [35]; in canned fish from Iraq, 0.0001 to 0.0003 mg·kg^−1^ [52]; in muscles from Iraq, 0.11–0.31 mg·kg^−1^ dry weight, in fresh fish 0.37–2.30 mg·kg^−1^ dry weight, in the muscles of frozen fish species and in canned fish 0.33–1.96 mg·kg^−1^ dry weight [42]; in canned tuna from the Kingdom of Saudi Arabia, 0.0018 mg·kg^−1^ for Ni [38]; in the canned fish samples, 0.58–1.04 mg·kg^−1^ [37]. In a study conducted in the USA, the content of Ni in canned fish was in the range from <LOQ to 0.783 mg·kg^−1^ [18]. Ni content in canned meat from Iraq was in the range from 0.0001 to 0.0007 mg·kg^−1^ [52], while the content of Ni in fish from Bangladesh was from 0.36 to 1.60 mg·kg^−1^ [44]. Brito et al. report that the content of Ni in various pork products from the European Union was from 0.49 mg·kg^−1^ to 10.63 mg·kg^−1^ [45]; the average content of Ni in beef from Nigeria was 0.25 mg kg^−1^ [48], in beef from Bangladesh 2.64–3.4 mg·kg^−1^ [53], in chicken meat < LOQ^−1^ 13 mg kg^−1^ [53], in carcass meat from UK–0.04 mg kg^−1^, and in poultry from UK–0.04 mg kg^−1^ [54]. The data on the content of Ni in canned fish obtained in this study, i.e., 0.088 mg kg^−1^, conform to the lower limits for the results available in the literature. In the case of canned meat, the concentration of Ni in this study was at the level of 0.004 mg·kg^−1^ and also conformed to the lower ranges of concentration described in the literature.

Cobalt is a component of vitamin B_12_ (cobalamin), a coenzyme which is essential in the formation of proteins, nucleic acids, and red blood corpuscles. Research has demonstrated that the percentage of cobalt absorbing in human body ranges from 20 to 97%. The largest amounts of cobalt are found in muscles, approx. 43%, and in bone tissue, approx. 14%. Excessive levels of the element are toxic and manifested in cardiac insufficiency and in hypothyroidism. In the diet, the content of cobalt ranged from 5 to 10 *μ*g per day. No daily intake requirement has been determined for this mineral, but it is assumed that the optimal dose should be not higher than 8 *μ*g per day [27]. According to literature data, the content of Co in canned fish from the USA was from 0.0 to 0.099 mg·kg^−1^ [18], which is close to our data (from 0.012 to 0.053 mg kg^−1^). The content of Co in meat products from Iraq was at the following levels: from 0.03 to 0.08 mg kg^−1^ (mean 0.48 mg kg^−1^) in canned corn beef and from 0.00 to 0.04 mg kg^−1^ (mean 0.02 mg kg^−1^) in canned chicken luncheon samples [47].

Silver occurs in nature in free state, but also in minerals. Silver is highly ductile and malleable; its thermal and electrical conductivity is the greatest among all metals. Metallic silver shows antibacterial and disinfecting properties. In the human body, silver does not play any biological role. An overdose of silver leads to cancer and a disease called argyria. The daily safe intake dose for humans is estimated at 1.8–80 mg. In normal conditions, humans are exposed to contact with silver every day, through food, water, and air. However, only 1–2% of the taken silver accumulates in the organism, and the rest is removed [55]. Ag content in fish from Bangladesh was found to be from <LOQ to 0.01 mg kg^−1^, which is lower than the level of that element in canned fish from the USA [44], from 0.0 to 0.197 mg kg^−1^ [18], which is similar to the data presented herein (from 0.011 to 0.0087 mg kg^−1^).

Vanadium is used in the production of catalysts, and it is also a structural material in the construction of nuclear reactors. In the human body it participates in the metabolism of hydrocarbons, hormones, and lipids. Vanadium has an insulinomimetic effect, stimulating the secretion of insulin in the pancreas. It participates in the mineralization of bones and regulates the metabolism of cysteine. The recommended daily intake of this element is from 50 *μ*g to 1 mg. In the case of type 2 diabetes, it was demonstrated that vanadium improves sensitivity to insulin, tolerance of glucose, and the concentration of total cholesterol [56]. Ikem and Egiebor (2005) report that the content of V in canned fish from the USA was from 0.0 to 0.312 mg kg^−1^ [18], which is comparable with the data presented in this paper, from 0.011 to 0.158 mg kg^−1^. Vanadium content in fish from Bangladesh was from 0.32 to 1.84 mg kg^−1^, which is higher than the concentration in analyzed canned fish [44].

Our study included additionally the determination of the content of antimony and uranium in canned meats and fishes. These elements are very rarely monitored in this group of products, which makes the discussion in this area very difficult.

Antimony in the form of inorganic compounds is more toxic than that in organic ones, and Sb (III) compounds are approximately 10 times more toxic than Sb (V) compounds. In turn, the toxicity of antimony compounds is about 10-fold lower than that of arsenic compounds. Elemental antimony is more toxic than its salts [57]. The biological role of antimony in the organism is not fully elucidated. The International Agency for Research on Cancer (IARC) stated that there is sufficient evidence from research on animals to accept that Sb_2_O_3_ is a carcinogenic compound [58]. The literature reports that the average content of Sb in fish from Xikuangshan (area of antimony mine, Hunan, China) was 0.218 mg kg^−1^ [59], while in this study the concentration of Sb found in samples of canned fish was in the range from 0.0014 to 0.0830 mg kg^−1^, i.e., at a considerably lower level. The content of Sb in fish from Bangladesh varied from 0.01 to 0.04 mg kg^−1^ and it was lower than the level of that element in analyzed canned fish [44].

Uranium is a radioactive element naturally occurring in various minerals and in magmatic rocks and can be present in water, air, food, and feed, at various concentrations, as a result of leaching from natural deposits (soils and rocks), emission from the nuclear industry, fallout of nuclear weapon testing, introduced with fertilisers, and combustion of coal and other fuels [60]. The content of U in fish from the Adriatic Sea was recorded so far at levels of 0.05–0.1 mg kg^−1^ [61], while the content of U in canned fish samples in the presented study was in the range from <LOQ to 0.226 mg kg^−1^.

The increasing level of consumption of meat and fish products is related to the improvement of the economic status of the population, and this affects the level of elements (especially toxic elements) consumed with food. Therefore, it is very important to assess health risk related to the consumption of products containing toxic elements.

### 3.3. Health Risk Assessment (Noncarcinogenic Risk)

The rank order of trace elements in canned fish of the THQ was as follows: As (up to 0.77576) > Cd (up to 0.11849) > Hg (up to 0.03394) > Sb (up to 0.03261) > Pb (up to 0.01292) > Co (up to 0.00042) > Ag (up to 0.00027) > Sn (up 0.00000) (Table 6). However, in the case of canned meat, the THQ was at a notably lower level: Cd (up to 0.01756) > Sb (up to 0.01164) > Pb (up to 0.00705) > Ag (up to 0.00120) > As (up to 0.00064) > Co (up to 0.00014) > Hg (up to 0.00002) > Sn (up 0.00000) (Table 6). This is because the concentration of As was higher than other toxic elements (Table 3) and also its RfD value was very low (0.0003 mg kg^−1^ d) [11]. THQ value of As was higher than other elements. TTHQ_max_ (due to tested products ingestion) was up to 0.97441 for canned fish and up to 0.03825 for canned meat (Table 6). Because the value of THQ ≤ 1, there is no probability of unfavorable effects occurring [9]. According to the literature data, the values of THQ for heavy metals were as follows: in fish from Bangladesh, Pb from 0.00 to 0.19, As from 0.01 to >1, Cd from 0.01 to >1 [62]; in canned tuna from Mexico, Pb up to 0.00027, Hg up to 0.1889, Cd up to 0.00003 [39]; in canned tuna from Italy, Pb up to 0.0043, Cd up to 0.102, Hg up to 1.441 [63]. Maximum values of THQ per individual albacore (*T. alalunga*), caught from NASSA and SASSA areas (Greece), for Hg, Cd, and Pb were respectively, 5.040, 0.359, and 0.075 [64].

After analyzing the data, we noticed that it is essential to carry out studies which could estimate the content of trace elements in food products that make up a significant contribution to the diet and may cause possible health problems.

The current state of knowledge does not allow for an unequivocal statement that the adopted toxicological standards are correct, especially since data on the effects of poisoning may appear after a long latency period. However, concluding the results obtained in the presented study, one can state that canned fish and meats produced in Poland should not pose any threat to human health in terms of toxic metal contents. In addition, the study shows that the meat products in particular, originating in their majority from local production, do not indicate any potential contamination of natural or agricultural areas with toxic elements.

## 4. Conclusions

The level of elements measured in different kinds of tested products was ranked as Mn > As > Cr > Sn > V > Ni > Pb > U > Sb > Co > Hg > Cd > Ag for canned fish, and Cr > Mn > Pb > Sn > Ag > Sb > Co > Cd > Ni > As > V > Hg > U for canned meat. It was shown that the rank order of heavy metals in canned fish based on THQ was As > Cd > Hg > Sb > Pb > Co > Ag > Sn. In the case of the canned meats, the THQ was at a considerably lower level, in the following rank order (decreasing values): Cd > Sb > Pb > Ag > As > Co > Hg > Sn. The value of THQ parameter for the analyzed elements in the canned foods was less than one, which means that there is no probability of unfavorable effects occurring. With reference to the applicable legal regulations regarding permissible levels of toxic elements in food [26], this study did not show that the concentration levels of these elements were exceeded.

## Figures and Tables

**Table 1:** Characteristics of the tested material.

Sample code	Meat or fish type	Number of samples analyzed	Composition and comment
Canned meat			
M1	Beef	3	Beef 65%, water, pork skins 10%, vegetable fiber, modified starch, salt, soy protein, milk protein, onion, black pepper, chili pepper, marjoram, ginger, phosphate stabilizers
M2	Beef	3	Beef 65%, water, pork skins 7%, bamboo fiber, modified starch, salt, monosodium glutamate, onion, vinegar, glucose, celery, phosphate stabilizers
M3	Beef	3	Beef 84%, water, modified corn starch, salt, spices, onions
M4	Beef	3	Beef 80%, water, pork skins 9%, pork lard, onions, salt, spices
M5	Beef	3	Beef 98%, cooking salt, spices
M6	Pork shoulder	3	Pork meat 76% (including pork shoulder meat 29%), water, salt, monosodium glutamate, maltodextrin, garlic, pepper, rapeseed oil, mustard, phosphate stabilizers, animal collagen protein
M7	Pork	3	Pork 90%, water, salt
M8	Pork stew	3	Pork 90%, water, salt
M9	Pork luncheon meat	3	Pork 93%, water, salt
M10	Pork luncheon meat	3	Pork 90%, water, salt
M11	Pork luncheon meat	3	Pork 91%, water, salt, pork rinds, garlic, spices, stabilizers: diphosphates
M12	Pork luncheon meat	3	Pork 93%, salt, stabilizers: phosphates, black pepper
M13	Pork pate	3	Pork meat 30%, water, pork fat, pork skins, pork liver 5%, modified starch, salt, pork heart, pork tongues, milk powder, wheat flour, onion, parsley, monosodium glutamate
M14	Chicken pate	3	Water, meat mechanically separated from chickens, rapeseed oil, liver and chicken skins, semolina, salt, soy protein, potato starch, onion, carrot, parsley, leek, herbal spices, milk powder, milk whey, sugar, maltodextrin, vegetable protein hydrolyzate, yeast extract

Canned fish			
F1	Tuna in sauce	3	Tuna (*Katsuwonus pelamis*) 64.8%, water, pineapple, pepper, sugar, vinegar, carrots, tomatoes, celery, bamboo, onion, tapioca starch, guar gum, thickeners, salt, pepper extract: natural dye, monosodium glutamate: flavor enhancer, garlic extract
F2	Tuna in oil	3	Tuna 65%, vegetable oil 20%, water, salt
F3	Tuna in sauce	3	Tuna (*Thunnus albacares*) 70%, water, salt
F4	Sardines in oil	3	Sardines 80%, vegetable oil, salt
F5	Sardines in oil	3	Sardines 70%, soybean oil, tabasco peppers, salt, vinegar
F6	Sardines in tomatoes	3	Sardines 66%, tomato sauce 35% and water, tomato concentrate, sugar, vegetable oil, salt, onion, herbal spice extract, guar gum, xanthan gum
F7	Smoked sprat in oil	3	Smoked sprat 60%, rapeseed oil 40%, salt
F8	Sprat in oil	3	Smoked sprat 60%, vegetable oil, salt
F9	Sprat in tomatoes	3	Sprat 68%, tomato sauce (32%) and water, tomato concentrate, sugar, spirit vinegar, vegetable oil, salt, onion, guar, locust bean gum, garlic, black pepper, allspice, sweet pepper, paprika aroma
F10	Herring in oil	3	Fillets of smoked herring 60%, rapeseed oil 40%, salt
F11	Herring in oil	3	Herring 50%, vegetable oil 46%, spices
F12	Herring in tomatoes	3	Herring 68%, tomato sauce 32% and water, tomato concentrate, sugar, spirit vinegar, vegetable oil, salt, onion, guar gum, locust bean gum, black pepper, paprika aroma
F13	Smoked mackerel in oil	3	Smoked mackerel fillet 60%, rapeseed oil 40%, salt
F14	Mackerel in tomatoes	3	Mackerel fillets 60%, tomato sauce 40% and water, 10% tomato concentrate, sugar, rapeseed oil, modified starch, spirit vinegar, salt, onion, herbal spice extract, guar gum, xanthan gum, paprika extract
F15	Brown bullhead in oil	3	Brown bullhead 60%, rapeseed oil 40%, salt
F16	Brown bullhead in tomatoes	3	Brown bullhead 50%, tomato sauce 50% and water, tomato concentrate, sugar, rapeseed oil, modified starch, spirit vinegar, salt, tomatoes, onion, extracts of vegetable spices, spices, guar gum, xanthan gum, paprika extract, maltodextrin

**Table 2:** The content of elements in the certified reference material: NIST-1577c Bovine Liver, NIST SRM 1548a, and TM 27.3 and validation parameters obtained during analysis.

Element	Certified reference material analysis		Validation parameters				
	The result declared by the manufacturer	The result obtained in own research	LOD	LOQ	Precision (%)	Recovery (%)	Uncertainty (%)
Co (mg kg^−1^)	0.300^a^	0.295	0.002	0.004	2.42	92	17
Ag (*μ*g kg^−1^)	5.9^a^	5.7	0.002	0.004	2.36	98	8
Sn (mg kg^−1^)	17.2^b^	16.40	0.021	0.042	7.43	96	18
Sb (*μ*g kg^−1^)	3.13^a^	3.28	0.023	0.079	1	104	7
Pb (*μ*g kg^−1^)	62.8^a^	62.2	0.005	0.01	6.07	99	12
Hg (*μ*g kg^−1^)	5.36^a^	5.21	0.0011	0.0015	3.80	90	24
Cd (*μ*g kg^−1^)	97.0^a^	96.3	0.004	0.007	2.42	99	6
As (*μ*g kg^−1^)	19.6^a^	20.4	0.004	0.009	3.18	101	7
Cr (*μ*g kg^−1^)	53^a^	55.2	0.028	0.055	2.40	100	8
Mn (mg kg^−1^)	10.46^a^	10.12	0.01	0.10	1	99	18
Ni (*μ*g kg^−1^)	44.5^a^	46.1	0.077	0.258	6.04	103	6
V (*μ*g kg^−1^)	8.17^a^	7.95	0.040	0.134	0.76	99	14
U (*μ*g kg^−1^)	2.03^c^	2.5	0.039	0.131	0.89	100	19

^a^NIST-1577c Bovine Liver, ^b^NIST SRM 1548a, ^c^TM 27.3.

**Table 3:** The trace elements (Co, Ag, Sn, Sb, Bi, Pb, Hg, Cd, As) contents of the analyzed canned meat and canned fish.

Code	Concentration (mg kg^−1^ product)							
	Co	Ag	Sn	Sb	Pb	Hg	Cd	As
Canned meat								
M1	0.017 ± 0.003ijkl	0.0038 ± 0.0011ghijk	0.055 ± 0.009fg	0.0112 ± 0.0024jkl	0.232 ± 0.033d	0.00004 ± 0.00001i	0.01983 ± 0.00076f	0.002 ± 0.000k
M2	0.006 ± 0.001m	0.0105 ± 0.0025f	0.006 ± 0.001i	0.0096 ± 0.0025kl	0.025 ± 0.006kl	0.00003 ± 0.00000ij	^*∗*^	0.002 ± 0.000k
M3	0.033 ± 0.007de	0.0660 ± 0.0121d	0.078 ± 0.018defg	0.0131 ± 0.0032jk	0.194 ± 0.032e	0.00003 ± 0.00001ij	^*∗*^	0.002 ± 0.000k
M4	0.043 ± 0.008bc	0.0741 ± 0.0128c	0.033 ± 0.007fg	0.0724 ± 0.0144b	0.350 ± 0.076b	0.00007 ± 0.00000i	^*∗*^	0.003 ± 0.000k
M5	0.015 ± 0.003ijkl	0.0039 ± 0.0008ghijk	0.174 ± 0.020cd	0.0157 ± 0.0031ij	0.206 ± 0.046de	0.00005 ± 0.00001i	0.00866 ± 0.00010h	0.002 ± 0.000k
M6	0.013 ± 0.002jklm	0.0794 ± 0.0131b	0.160 ± 0.019cde	0.0579 ± 0.0111cd	0.201 ± 0.054de	0.00002 ± 0.00000j	^*∗*^	0.003 ± 0.000k
M7	0.010 ± 0.001lm	0.0089 ± 0.0019fg	0.029 ± 0.010gh	0.0028 ± 0.0006n	0.106 ± 0.024hi	0.00004 ± 0.00000i	0.00509 ± 0.00038ij	0.002 ± 0.000k
M8	0.012 ± 0.002klm	0.0520 ± 0. 0101e	0.018 ± 0.005h	0.0390 ± 0.0095f	0.153 ± 0.034f	0.00004 ± 0.00000i	^*∗*^	0.002 ± 0.000k
M9	0.005 ± 0.001m	0.0094 ± 0. 0012fg	0.005 ± 0.001i	0.0095 ± 0.0017kl	0.013 ± 0.004l	0.00002 ± 0.00000j	^*∗*^	0.002 ± 0.000k
M10	0.005 ± 0.002m	0.0554 ± 0.0111e	0.015 ± 0.005h	0.0089 ± 0.0012klm	0.147 ± 0.041fg	0.00003 ± 0.00000ij	^*∗*^	0.002 ± 0.000k
M11	0.022 ± 0.005fghi	0.0045 ± 0.0005fg	0.068 ± 0.011efg	0.0040 ± 0.0008mn	0.395 ± 0.066a	0.00003 ± 0.00001ij	0.02731 ± 0.00021d	0.002 ± 0.000k
M12	0.033 ± 0.006de	0.0936 ± 0.0215e	0.096 ± 0.027defg	0.0702 ± 0.0127b	0.217 ± 0.041de	0.00003 ± 0.00000ij	^*∗*^	0.002 ± 0.000k
M13	0.010 ± 0.002lm	0.0046 ± 0.0009ghijk	0.034 ± 0.007fg	0.0061 ± 0.0017lmn	0.349 ± 0.078b	0.00004 ± 0.00001i	0.00860 ± 0.00015h	0.002 ± 0.000k
M14	0.027 ± 0.005efgh	0.0743 ± 0.0134c	0.052 ± 0.013fg	0.0547 ± 0.0109de	0.238 ± 0.049d	0.00001 ± 0.00000j	^*∗*^	0.002 ± 0.000k
Mean	0.018B	0.0386A	0.059B	0.0268A	0.202A	0.00003B	0.00496B	0.002B

Canned fish								
F1	0.022 ± 0.006fghi	0.0078 ± 0.0016fgh	0.120 ± 0.011cdef	0.0036 ± 0.0008n	0.014 ± 0.005l	0.06180 ± 0.00910b	0.0644 ± 0.0133b	0.963 ± 0.290ef
F2	0.050 ± 0.017ab	0.0069 ± 0.0015fghij	0.095 ± 0.019defg	0.0040 ± 0.0015mn	0.296 ± 0.073c	0.03690 ± 0.00310c	0.0088 ± 0.0014h	0.709 ± 0.060g
F3	0.016 ± 0.004ijkl	0.0018 ± 0.0009jk	0.018 ± 0.005h	0.0280 ± 0.0071gh	0.035 ± 0.011kl	0.07840 ± 0.01050a	0.0754 ± 0.0117a	0.897 ± 0.271f
F4	0.030 ± 0.009def	0.0045 ± 0.0012ghijk	0.069 ± 0.014efg	0.0830 ± 0.0210a	0.039 ± 0.013kl	0.01250 ± 0.00120efgh	0.0085 ± 0.0024h	1.481 ± 0.394a
F5	0.035 ± 0.011de	0.0087 ± 0.0016fg	0.073 ± 0.017defg	0.0250 ± 0.0060h	0.037 ± 0.015kl	0.01770 ± 0.00210efg	0.0174 ± 0.007g	1.181 ± 0.322b
F6	0.021 ± 0.005ghij	0.0045 ± 0.0014ghijk	0.744 ± 0.061b	0.0111 ± 0.0032jkl	0.021 ± 0.006kl	0.00820 ± 0.00270ghi	0.0033 ± 0.0016j	0.709 ± 0.191g
F7	0.027 ± 0.009efgh	0.0029 ± 0.0012hijk	0.069 ± 0.021efg	0.0014 ± 0.0025o	0.019 ± 0.005kl	0.01280 ± 0.00260efgh	0.0335 ± 0.0093c	1.094 ± 0.256cd
F8	0.027 ± 0.008efgh	0.0061 ± 0.0024fghijk	0.055 ± 0.015fg	0.0572 ± 0.0121cd	0.091 ± 0.015hij	0.01190 ± 0.00350efghi	0.0211 ± 0.0055f	0.496 ± 0.147i
F9	0.053 ± 0.014a	0.0011 ± 0.0003k	0.042 ± 0.012fg	0.0193 ± 0.0072hij	0.036 ± 0.011kl	0.02100 ± 0.00480de	0.0047 ± 0.0012ij	1.187 ± 0.368b
F10	0.019 ± 0.005hijk	0.0059 ± 0.0013fghijk	0.034 ± 0.014fg	0.0420 ± 0.0112f	0.014 ± 0.004kl	0.06480 ± 0.01270b	0.0235 ± 0.0056e	0.686 ± 0.174g
F11	0.028 ± 0.012efg	0.0050 ± 0.0019fghijk	0.081 ± 0.021defg	0.0507 ± 0.0131de	0.075 ± 0.016ij	0.01510 ± 0.00260efgh	0.0241 ± 0.0067e	0.704 ± 0.158g
F12	0.037 ± 0.014cd	0.0079 ± 0.0025fgh	0.072 ± 0.017defg	0.0740 ± 0.0214b	0.109 ± 0.034hi	0.00910 ± 0.00160fghi	0.0154 ± 0.0036g	0.577 ± 0.184h
F13	0.012 ± 0.003klm	0.0051 ± 0.0017fghijk	0.210 ± 0.054c	0.0531 ± 0.0161de	0.011 ± 0.003l	0.03080 ± 0.00780cd	0.0089 ± 0.0017h	1.036 ± 0.234de
F14	0.017 ± 0.004ijkl	0.0074 ± 0.0027fghi	0.086 ± 0.027defg	0.0620 ± 0.0098c	0.120 ± 0.035fgh	0.02080 ± 0.00430de	0.0037 ± 0.0012j	1.166 ± 0.351bc
F15	0.029 ± 0.012efg	0.0066 ± 0.0021fghijk	0.064 ± 0.019efg	0.0580 ± 0.0067cd	0.112 ± 0.031ghi	0.02020 ± 0.00380def	0.0033 ± 0.0010j	0.462 ± 0.126i
F16	0.034 ± 0.14de	0.0020 ± 0.0007ijk	1.362 ± 0.254a	0.0310 ± 0.0072g	0.057 ± 0.013jk	0.00610 ± 0.00070hi	0.0067 ± 0.0012hi	0.359 ± 0.082j
Mean	0.028A	0.0053B	0.200A	0.0377A	0.068B	0.02676A	0.0202A	0.857A

^*∗*^Below the limit of quantitation (<LOQ)—Table 2. Values designated with the same letters (a, b, c, ..., A, B) within column do not significantly differ at 5% error (Duncan's test).

**Table 4:** The V, Cr, Mn, Ni, and U contents of the analyzed canned meat and canned fish.

Code	Concentration (mg kg^−1^ product)				
	V	Cr	Mn	Ni	U
Canned meat					
M1	^*∗*^	0.168 ± 0.032m	0.195 ± 0.051fg	0.004 ± 0.001l	^*∗*^
M2	^*∗*^	0.215 ± 0.024lm	0.223 ± 0.058fg	0.003 ± 0.001l	^*∗*^
M3	^*∗*^	0.243 ± 0.064kl	0.167 ± 0.039fg	0.004 ± 0.002l	^*∗*^
M4	0.0003 ± 0.0001i	0.259 ± 0.059jkl	0.129 ± 0.027g	0.002 ± 0.001l	^*∗*^
M5	0.0004 ± 0.0001i	0.219 ± 0.034klm	0.143 ± 0.032fg	^*∗*^	^*∗*^
M6	0.0004 ± 0.0001i	0.287 ± 0.075jk	0.164 ± 0.038fg	0.003 ± 0.001l	^*∗*^
M7	0.0004 ± 0.0001i	0.246 ± 0.058kl	0.215 ± 0.061fg	^*∗*^	^*∗*^
M8	0.0008 ± 0.0002i	0.325 ± 0.084ij	0.178 ± 0.035fg	0.008 ± 0.003kl	^*∗*^
M9	^*∗*^	0.214 ± 0.026lm	0.156 ± 0.034fg	^*∗*^	^*∗*^
M10	0.0009 ± 0.0003i	0.374 ± 0.092i	0.234 ± 0.068fg	0.012 ± 0.003k	^*∗*^
M11	^*∗*^	0.283 ± 0.068jkl	0.247 ± 0.075fg	^*∗*^	^*∗*^
M12	^*∗*^	0.247 ± 0.062kl	0.195 ± 0.047fg	0.007 ± 0.002kl	^*∗*^
M13	0.0003 ± 0.0001i	0.172 ± 0.035m	0.147 ± 0.037fg	0.004 ± 0.001l	^*∗*^
M14	^*∗*^	0.158 ± 0.029m	0.624 ± 0.152de	^*∗*^	^*∗*^
Mean	0.0003B	0.244B	0.216B	0.004B	^*∗*^

Canned fish					
F1	0.114 ± 0.022cde	0.543 ± 0.127efgh	0.137 ± 0.036fg	0.032 ± 0.007j	0.226 ± 0.053a
F2	0.122 ± 0.018bc	0.719 ± 0.270a	0.205 ± 0.055fg	0.079 ± 0.023g	^*∗*^
F3	0.055 ± 0.012h	0.608 ± 0.180cde	0.141 ± 0.034fg	0.048 ± 0.013i	^*∗*^
F4	0.137 ± 0.027b	0.507 ± 0.105gh	1.965 ± 0.612b	0.052 ± 0.017i	^*∗*^
F5	0.158 ± 0.044a	0.562 ± 0.101defgh	2.566 ± 0.724a	0.073 ± 0.019g	^*∗*^
F6	0.118 ± 0.021bcd	0.614 ± 0.171cd	1.302 ± 0.310c	0.047 ± 0.012i	^*∗*^
F7	0.052 ± 0.019h	0.572 ± 0.116defg	2.050 ± 0.591b	0.120 ± 0.029d	0.134 ± 0.027b
F8	0.134 ± 0.032b	0.573 ± 0.121defg	1.343 ± 0.301c	0.089 ± 0.018f	0.023 ± 0.006cd
F9	0.048 ± 0.009h	0.587 ± 0.111cdef	2.809 ± 0.824a	0.128 ± 0.032c	^*∗*^
F10	0.097 ± 0.022ef	0.500 ± 0.091h	1.253 ± 0.325c	0.101 ± 0.021e	0.135 ± 0.031b
F11	0.137 ± 0.029b	0.646 ± 0.178bc	0.810 ± 0.265de	0.089 ± 0.028f	0.020 ± 0.006cd
F12	0.074 ± 0.021g	0.528 ± 0.123fgh	1.923 ± 0.620b	0.144 ± 0.044b	0.027 ± 0.007c
F13	0.088 ± 0.019fg	0.725 ± 0.221a	0.674 ± 0.243de	0.062 ± 0.015h	0.142 ± 0.031b
F14	0.078 ± 0.022g	0.532 ± 0.091fgh	0.515 ± 0.155ef	0.080 ± 0.021g	0.026 ± 0.007c
F15	0.100 ± 0.027def	0.695 ±.185ab	0.518 ± 0.149ef	0.106 ± 0.026e	0.017 ± 0.0004cd
F16	0.011 ± 0.003i	0.533 ± 0.131fgh	0.923 ± 0.189d	0.162 ± 0.042a	^*∗*^
Mean	0.095A	0.590A	1.196A	0.088A	0.047A

^*∗*^Below the limit of quantitation (<LOQ)—Table 2. Values designated with the same letters (a, b, c, ..., A, B) within column do not significantly differ at 5% error (Duncan's test).

**Table 5:** Comparison of obtained data on the content of elements in fish and in canned meat and fish with data from the literature and with the maximum acceptable residual level.

Concentration (mg kg^−1^ product)			
Element	Experimental data	Literature data	Maximum acceptable residual level
Canned meat			
Hg	0.00001–0.00007	0.001–2 [52]; 0.125 in d.w. [46]; <LOQ-0.840, <LOQ-0.900, <LOQ-0.980, <LOQ-1.220, [65]	—
As	0.002–0.003	< LOQ [66]	0.20 [67]
Cd	<LOQ-0.02731	1 [66]; 4.08 d.w. [46]; 0.06–0.27 (mean 0.60) and 0.06–0.10 (mean 0.08) [47]; 0.020–0.220 [45]; <LOQ-0.110, <LOQ-0.100, <LOQ-0.090 [65]	0.05 [26], 0.50 [68]
Pb	0.013–0.395	4–4.20 [66]; 15.43 in d.w. [46]; 0.53–2.07 (mean 1.3) 0.52–1.19 (mean 0.85) [47]; 0.15–2.06 [45]; <LOQ-1.14, <LOQ-0.630, <LOQ-0.830 [65]	0.10 [26], 0.50 [26], 0.50 [69], [70]
Sn	0.005–0.174	<LOQ-6.220, <LOQ-6.350, <LOQ-5.280, <LOQ-5.460 [65]	200 [26], 250 [71]

Fish^*∗*^ and canned fish			
Hg	0.00610–0.07840	0.043–0.253 [16]; 0.18–0.86 [41]; 0.053–0.7396, 0.0195–0.0489 [18]; 0.07–0.22 [72]; <LOQ-1.14 [17]; 0.0001–0.0003 [52]; 0.0378–0.5243 [73]; 0.60–0.62 [74]; 0.088–0.410, [43]; 0.005–1.17 [39], ^*∗*^0.140–0.327 in d.w. [75], ^*∗*^0.094–0.466 [76], ^*∗*^0.16–0.53 [77]	0.50 and 1.0 (tuna) [26]
As	0.359–1.481	0.0369–0.2618 [16]; <LOQ-1 [66]; 0.49–25.26 [35]; <LOQ-1.72, <LOQ-0.15 [18]; 1.03–1.41 [72], ^*∗*^0.004–0.073 in d.w. [75], ^*∗*^0.105–0.333 [76], ^*∗*^0.003–0.08 [77]	4 [67]
Cd	0.0033–0.0754	0.0046–0.0720 [16]; 0.002–0.07 [78]; 1.00–1.80 [66]; 0.08–0.66 [41]; 0.0–53.9 [18]; 0.019 [79]; <LOQ-0.09 [17]; 0.08 [36]; 0.0032–0.0834 [73]; 0.020–0.025 [74]; <LOQ-0.007 [39]; 0.03–0.12 [37]; <LOQ-0.18 [40]; 0.19–17.05 [80], ^*∗*^0.008–0.191 in d.w. [75], ^*∗*^0.021–0.082 [76], ^*∗*^0.01–0.21 [77]	0.050 and 0.10 (sardine, tuna) [26]
Pb	0.011–0.296	0.0126–0.0726 [16]; 0.007–0.51 [78]; 3–4.60, 3.60–4.00 [66]; 0.14–0.92 [41]; 0.02–1.37 [35]; 0.0–31.1 [18]; 0.11 [79]; <LOQ-4.13 [17]; 0.10 [36]; <LOQ-3.19, 0.35–2.10, 0.25–1.51 in d.w. [42]; 0.0043–0.0856 [73]; 0.011–0.089 [74]; 0.058–0.168 [43]; 0.07–0.32, 0.04–0.32 [39]; 0.18–0.38 [37]; 0.05–0.34 [40]; 0.12–78.42 [80], ^*∗*^0.014–1.518 in d.w. [75], ^*∗*^0.019–0.084 [76], ^*∗*^0.048–1.30 [77]	0.30 [26]
Sn	0.018–1.362	0.023–13.108 [78]; 0.14–4.03 [35]; 0.15–6.34 [40]; 0.04–28.7 [18]	200 [26]

^*∗*^Data for the fish tissues.

**Table 6:** The health risk assessment related to the consumption of canned meat and canned fish.

	Co	Ag	Sn	Sb	*P*b	Hg	Cd	As
^*∗*^THQ^1^								
Canned meat (mean)	0.00006	0.00050	0.00000	0.00431	0.00361	0.00001	0.00319	0.00043
Canned meat (max)	0.00014	0.00120	0.00000	0.01164	0.00705	0.00002	0.01756	0.00064
Canned fish (mean)	0.00022	0.00017	0.00000	0.01481	0.00297	0.01402	0.03174	0.44875
Canned fish (max)	0.00042	0.00027	0.00000	0.03261	0.01292	0.03394	0.11849	0.77576

^*∗∗*^TTHQ^1^								
Canned meat (mean)	0.01209							
Canned meat (max)	0.03825							
Canned fish (mean)	0.51267							
Canned fish (max)	0.97441							

^*∗∗∗*^EDI^1^								
Canned meat (mean)	0.00116	0.00248	0.00379	0.00000	0.01299	0.00000	0.00032	0.00013
Canned meat (max)	0.00276	0.00602	0.01029	0.00000	0.02539	0.00000	0.00176	0.00019
Canned fish (mean)	0.00440	0.00083	0.03143	0.00001	0.01069	0.00421	0.00317	0.13462
Canned fish (max)	0.00833	0.00137	0.21403	0.00001	0.04651	0.01018	0.01185	0.23273

^*∗*^THQ^2^								
Canned meat (mean)	0.00016	0.00135	0.00000	0.01173	0.00982	0.00002	0.00868	0.00117
Canned meat (max)	0.00038	0.00328	0.00000	0.03168	0.01920	0.00004	0.04780	0.00175
Canned fish (mean)	0.00010	0.00007	0.00000	0.00652	0.00131	0.00617	0.01398	0.19761
Canned fish (max)	0.00018	0.00012	0.00000	0.01436	0.00569	0.01495	0.05218	0.34161

^*∗∗*^TTHQ^2^								
Canned meat (mean)	0.03292							
Canned meat (max)	0.10413							
Canned fish (mean)	0.22576							
Canned fish (max)	0.42908							

^*∗∗∗*^EDI^2^								
Canned meat (mean)	0.01106	0.02371	0.03624	0.01646	0.12409	0.00002	0.00305	0.00123
Canned meat (max)	0.02641	0.05750	0.09829	0.04447	0.24264	0.00004	0.01678	0.00184
Canned fish (mean)	0.00680	0.00129	0.04857	0.00916	0.01651	0.00650	0.00491	0.20806
Canned fish (max)	0.01287	0.00211	0.33077	0.02016	0.07189	0.01574	0.01831	0.35967

^*∗*^THQ: target hazard quotient, ^*∗∗*^TTHQ: total target hazard quotient, ^*∗∗∗*^EDI: estimated daily intake. ^1^Average daily consumption of canned products in Poland: canned meat, 4.5 g·day^−1^ (which corresponds to a portion of about 0.1 canned meat items weighing 300 g·week^−1^); canned fish, 11 g·day^−1^ (which corresponds to a portion of about 0.6 canned fish items weighing 120 g·week^−1^); exposure duration, 70 years. ^2^Daily consumption of canned meat, 43 g·day^−1^ (one canned meat item with a product content of 300 g·week^−1^); canned fish, 17 g·day^−1^ (one canned fish item with a product content of 120 g·week^−1^); exposure duration, 30 years.

## Data Availability

The data used to support the findings of this study are included within the article.
